# Filler rhinoplasty based on anatomy: The dual plane technique

**DOI:** 10.1016/j.jpra.2019.04.002

**Published:** 2019-04-17

**Authors:** Gyu Sik Jung

**Affiliations:** GLAD Plastic Surgery Clinic, 31 Dongsung-ro 5-gil, 3rd floor, Jung-gu, Daegu 41941, Republic of Korea

**Keywords:** Dual plane, Filler, Rhinoplasty, Hyaluronic acid

## Abstract

Filler rhinoplasty is susceptible to spreading because of the way the nose protrudes away from the face. Often, the well-defined appearance immediately after the procedure is poorly maintained, and the nasion area develops a bulbous appearance. Therefore, I developed a “dual plane technique” to prevent spreading and to maintain the desired shape of the filler rhinoplasty. Dual plane injections were administered in 96 patients. Based on the anatomy of the nose, a highly elastic filler was injected in the deep fat layer and a moderately elastic filler was injected in the superficial fat compartments. The retrograde linear threading technique was used to inject the fillers to prevent mixing with each other. Afterwards, the highly elastic hyaluronic acid filler was injected from the anterior nasal spine into the supracartilaginous layer by a retrograde linear threading technique. All patients were followed up for at least 3 months, during which time there were no major complications and aesthetic satisfaction was high. The dual plane technique is an injection technique based on the anatomical base. Different types of filler can be used according to the plane and filler rheology to obtain an aesthetically pleasing result.

## Introduction

Rhinoplasty is one of the most common aesthetic procedures performed by plastic surgeons worldwide. In recent times, a non-surgical rhinoplasty has become the preferred option for most patients and physicians.^1^ The most common non-surgical method for volume correction is fillers.^2^ Filling properties allow the physician to obtain optimal results with a minimal downtime and good longevity.^3^ In the past, fillers were used for surface treatments with a dermatologic approach and temporary results. Presently, fillers are no longer used to address wrinkles but mostly to restore volumes.^2^ In fact, fillers are injected into the deep planes and not the skin; the injection strategy is based on surgical concepts and is aimed at maintaining the results.^4^ The soft tissue of the nose consists of five layers: the skin, superficial fatty layer, fibromuscular layer, deep fatty layer and the periosteum/perichondrium.^1^ I injected fillers into two separate layers: the superficial fatty later and the deep fatty layer. I named this strategy as the ‘dual plane technique’. Through this report, I want to share my experiences with the dual plane technique using a hyaluronic acid filler and describe the aesthetic outcomes and the associated complications.

## Methods

Between January and September 2018, 96 patients who underwent the dual plane technique with a hyaluronic acid filler were included in the study. Clinical and demographic features of the patients, the technique, satisfaction rates and complications were noted. Satisfaction was investigated immediately after the procedure, and all patients were followed up for 3 months. The satisfaction survey was repeated 3 months after the procedure. The satisfaction score was assigned using a questionnaire with a scale ranging from 1 to 5: 1—dissatisfied, 2—minimal satisfaction, 3—moderate satisfaction, 4—high satisfaction and 5—full satisfaction. Photos were taken before the injection, immediately after and 3 months later. All procedures performed in this study were in accordance with the ethical standards of the institutional and/or national research committee and with the 1964 Helsinki Declaration and its later amendments or comparable ethical standards. Informed consent was obtained before any surgical procedures and for photography purposes.

### Surgical procedure of the dual plane technique

**Figure 1:**
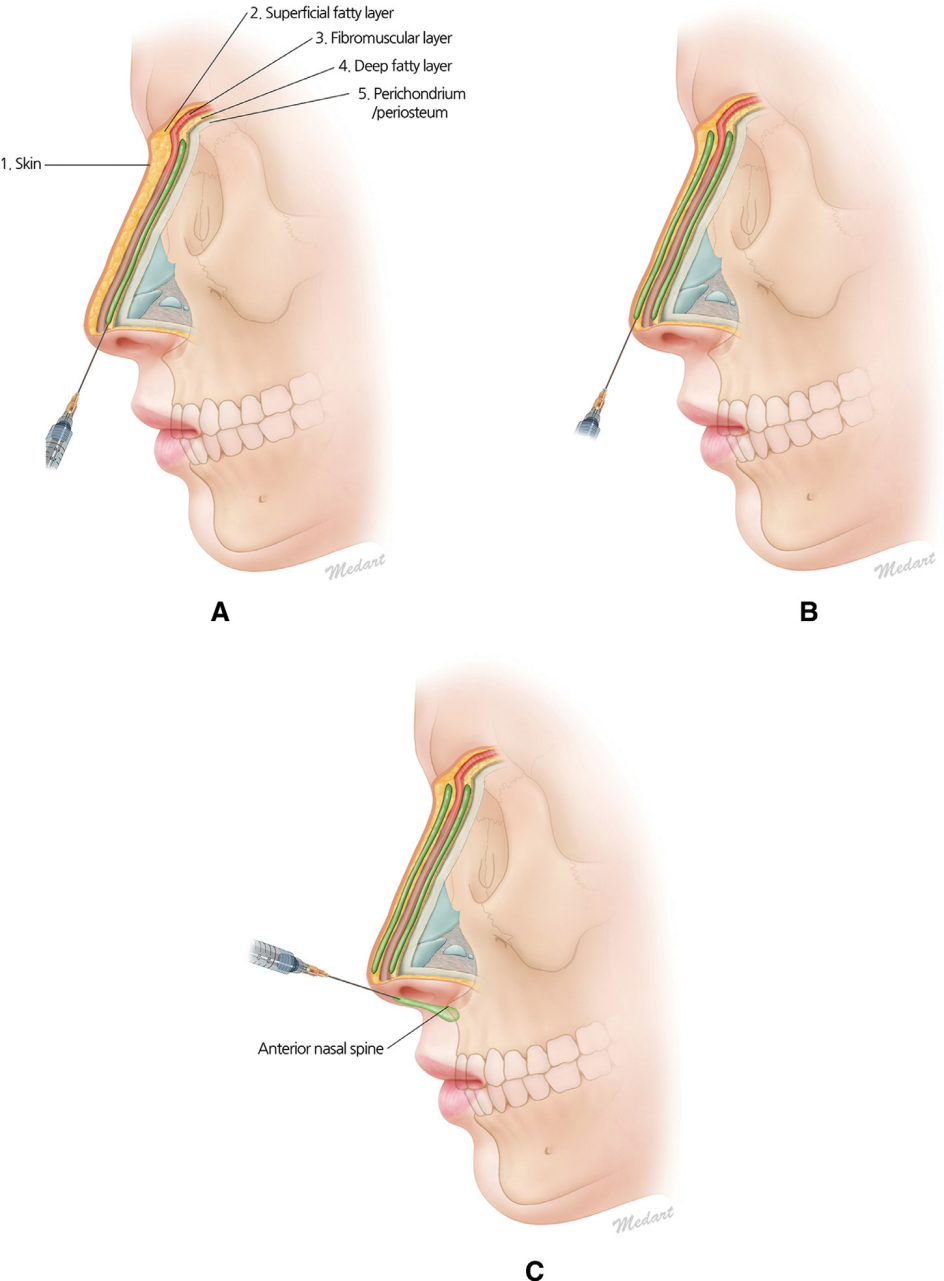
Schematic illustration of the dual plane technique. A. Deep fatty layer injection. B. Superficial fatty layer injection. C. Nasal tip injection.

A nerve block was applied to the infratrochlear nerve and internal nasal branch of infraorbital nerve using 1% lidocaine with 1:200,000 epinephrine, and the procedure was initiated 10 minutes later.^5^ I used dihydrochloride to disinfect the skin. First, the highly elastic hyaluronic acid filler (Teosyal**^®^** PureSense Ultra Deep, Teoxane Lab, Switzerland) was injected into the deep fatty layer. A 25 gauge and a 70 mm blunt cannula were used for the filler injection. The entry point was made with a 25 gauge sharp needle on the infratip lobule. The cannula was slowly moved through the deep fatty layer until it reached the radix. The skin of the nasal dorsum was gently lifted with the thumb and a second finger during the cannula movement. After the cannula reached the radix, the filler was injected into the nasal dorsum in the deep fatty layer by a retrograde linear threading technique. Next, a cannula of the same size was used at the same entry point to inject the moderately elastic hyaluronic acid filler (Teosyal**^®^** PureSense Ultimate, Teoxane Lab, Switzerland) in the superficial fatty layer. The cannula was slowly moved forward in the superficial fatty layer until it reached the radix. The filler was injected into the nasal dorsum in the superficial fatty layer by a retrograde linear threading technique. Afterwards, the same cannula was inserted perpendicular to the nasal tip through the same entry hole. Subsequently, it was gently moved forward from the tip to the columella and nasolabial angle. After the cannula reached the anterior nasal spine, the highly elastic hyaluronic acid filler (Teosyal**^®^** PureSense Ultra Deep) was injected into the supracartilaginous layer by a retrograde linear threading technique. Finally, the cannula was removed from the tip of the nose. The amount of hyaluronic acid filler used ranged from 0.4 to 1.2 mL (Figure 1).

## Results

**Figure 2:**
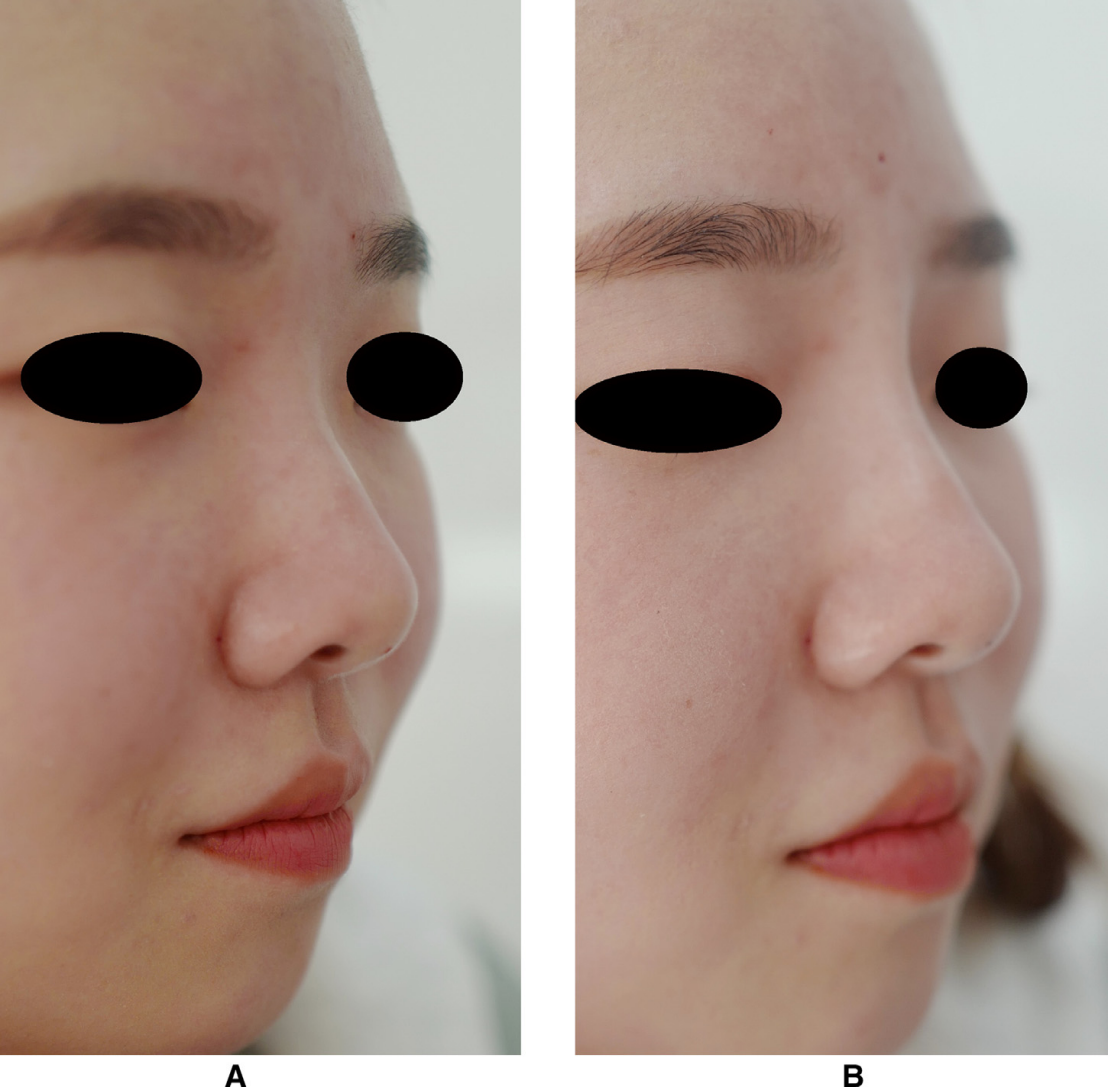
Photographs of a 27-year-old woman. A. Preoperatively. B. 3 months postoperatively.

**Figure 3:**
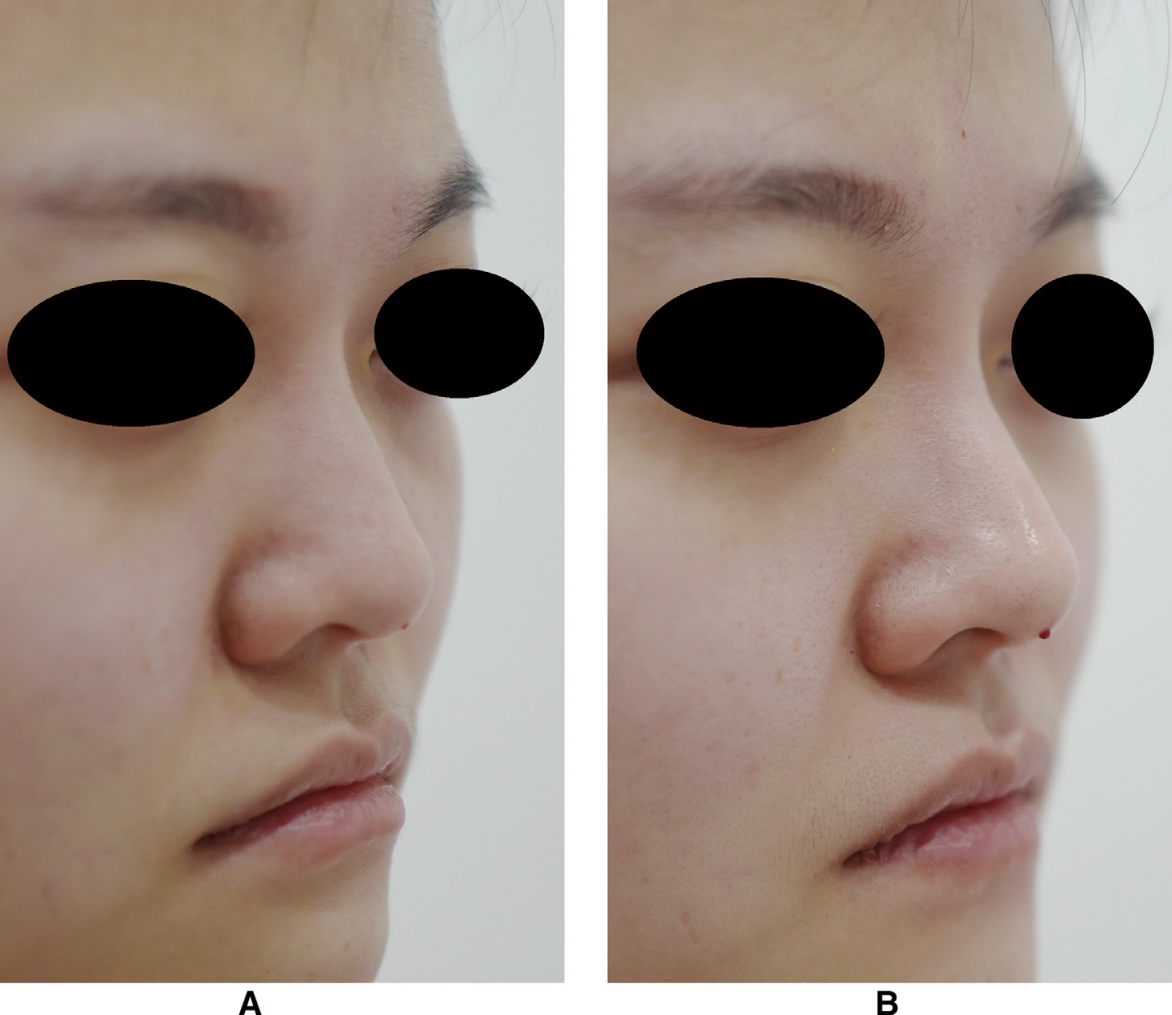
Photographs of a 36-year-old woman. A. Preoperatively. B. 3 months postoperatively.

Between January and September 2018, 96 patients (89 women and 7 men) were treated with the dual plane technique using a hyaluronic acid filler. The mean age of the patients was 33.8 years (range: 22–48). None of the patients underwent prior nose surgery or a non-surgical nose job, nasal obstruction or nasal deformity. The mean patient satisfaction scores were 4.8 ± 0.8 (standard deviation) points immediately after the operation and 4.7 ± 0.7 points at 3 months postoperatively. No serious adverse events occurred during the course of the treatment. Two patients experienced temporary mild erythema, and two patients had mild ecchymosis after the treatment, with complication resolution 3 days later and healing without sequelae. Nevertheless, a very high patient satisfaction rate was noted in the study. The patients were informed that the results could last approximately 12 months after the filler injection. Patients’ follow-up showed an acceptable temporary nasal deformity correction and near-normal nasal aesthetic angles, up to 3 months, which camouflaged the dorsal hump and nasal length. The frontonasal, nasolabial and nasofacial angles were markedly improved and were near the normal measurements. Saddle-nose deformity, supratip depression, infratip lobule depression, alar irregularities, domal definition and columellar lengthening were corrected. In particular, there was no swelling of the nasal bridge (Figure 2, Figure 3).

## Discussion

**Figure 4:**
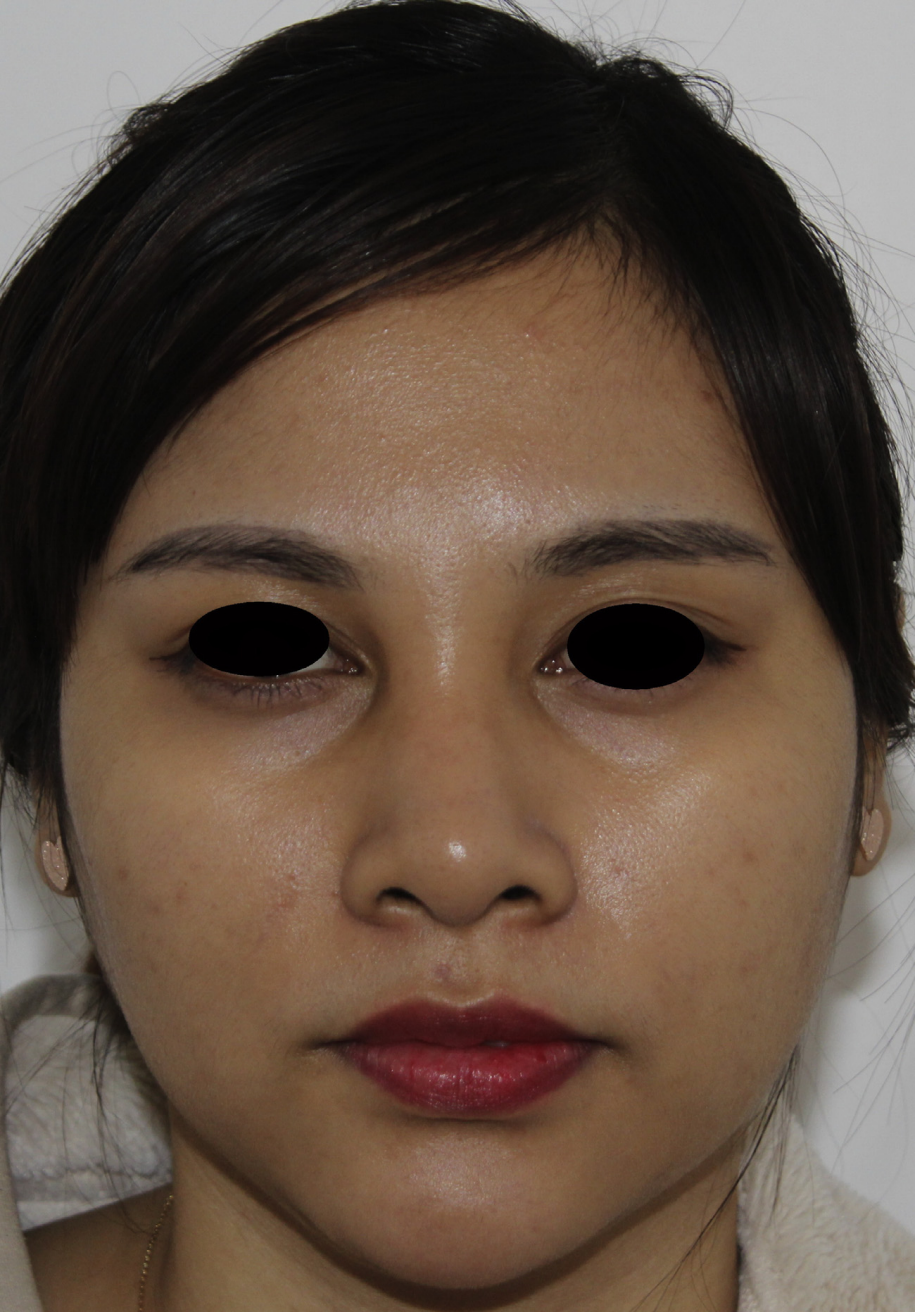
‘Avatar’ nose. Spreading of the filler in the glabellar region can sometimes create the appearance of a so-called ‘Avatar’ nose.

After a filler rhinoplasty by the single layer technique, spreading of the filler in the glabellar region can sometimes create the appearance of a so-called ‘Avatar’ nose (Figure 4). Certain individuals may develop swelling due to inflammation after the procedure, while in some cases, the filler, being a gel, may spread into the nasal bridge and alae during the process of absorption. In this study, I was able to reduce filler spread by the dual plane technique, hence achieving a longer nose with a reduced widening than that by the single layer technique. The major blood vessels of the nose are known to be present in the superficial fatty layer, fibromuscular layer and deep fatty layer.^1^, ^6^ Therefore, in filler rhinoplasty, it is safer to administer injections through a cannula rather than a needle. Because of the fact that using a cannula can cause vessel damage complications,^7^ I advise that the procedure should be performed slowly, by injecting the filler using the retrograde linear technique.^8^ It has been reported that the major blood vessels are relatively absent in the midline 3 mm of the nose; therefore, the surgeon should mark the midline on the nasal bridge and, to minimise asymmetry, perform the procedure without deviating from it. Awareness towards the risk of blindness should be crucial, and I believe performing this procedure slowly is the most important aspect in reducing complications.^9^, ^10^ The dual plane technique is an injection technique based on the anatomical base. Different types of fillers can be used according to the plane of injection and their rheology to obtain a successful aesthetic result.

## Patient consent

Patients provided written consent for the use of their images.

## Appendix Supplementary materials

Image, application 1
